# An assay for the identification of *Plasmodium simium* infection for diagnosis of zoonotic malaria in the Brazilian Atlantic Forest

**DOI:** 10.1038/s41598-017-18216-x

**Published:** 2018-01-08

**Authors:** Denise Anete Madureira de Alvarenga, Richard Culleton, Anielle de Pina-Costa, Danielle Fonseca Rodrigues, Cesare Bianco, Sidnei Silva, Ana Júlia Dutra Nunes, Julio César de Souza, Zelinda Maria Braga Hirano, Sílvia Bahadian Moreira, Alcides Pissinatti, Filipe Vieira Santos de Abreu, André Luiz Lisboa Areas, Ricardo Lourenço-de-Oliveira, Mariano Gustavo Zalis, Maria de Fátima Ferreira-da-Cruz, Patricia Brasil, Cláudio Tadeu Daniel-Ribeiro, Cristiana Ferreira Alves de Brito

**Affiliations:** 1 Grupo de Pesquisa em Biologia Molecular e Imunologia da Malária, Instituto René Rachou (IRR), Fundação Oswaldo Cruz (Fiocruz), Belo Horizonte/MG, 30190-009 Brazil; 2 Malaria Unit, Department of Pathology, Institute of Tropical Medicine, Nagasaki University, Nagasaki, 852-8523 Japan; 3 Laboratório de Doenças Febris Agudas, Instituto Nacional de Infectologia Evandro Chagas (INI), Fiocruz, Rio de Janeiro/RJ 21040-360 Brazil; 4 Centro de Pesquisa, Diagnóstico e Treinamento em Malária (CPD-Mal), Fiocruz, Rio de Janeiro/RJ 21040-360 Brazil; 5 Laboratório de Pesquisa em Malária, Instituto Oswaldo Cruz (IOC), Fiocruz, Rio de Janeiro/RJ 21040-360 Brazil; 6 Núcleo de Educação Ambiental Fábio Perini, Perini Business Park, Joinville/SC, 89219-600 Brazil; 7 Centro de Pesquisas Biológicas de Indaial, Indaial/SC, 89130-000 Brazil; 8 Universidade Regional de Blumenau – FURB, Blumenau/SC, 89012-900 Brazil; 9 Centro de Primatologia do Rio de Janeiro (CPRJ/INEA), Guapimirim/RJ, 25940-000 Brazil; 10 Centro Universitário Serra dos Órgãos (UNIFESO), Teresópolis/RJ, 25964-004 Brazil; 11 Laboratório de Mosquitos Transmissores de Hematozoários, Fiocruz, Rio de Janeiro/RJ 21040-360 Brazil; 12 Laboratório de Infectologia e Parasitologia Molecular, Hospital Universitário Clementino Fraga Filho, Universidade, Federal do Rio de Janeiro/RJ 21941-901 Brazil

**Keywords:** Diagnostic markers, Malaria

## Abstract

Zoonotic malaria poses a unique problem for malaria control. Autochthonous cases of human malaria in the Atlantic Forest have recently been attributed to *Plasmodium simium*, a parasite that commonly infects non-human primates in this Brazilian biome. However, due to its close similarity at both the morphological and molecular level to *Plasmodium vivax*, the diagnosis of *P. simium* in this region remains problematic. Therefore, a diagnostic assay able to accurately identify *P. simium* is important for malaria surveillance. Based on mitochondrial genome sequences, primers were designed to amplify a region containing a SNP specific to *P. simium*. This region can then be digested with the restriction enzyme *Hpy*CH4III, which results in digestion of *P. simium* sequences, but not of any other malaria parasite. Fifty-two human and monkey blood samples from different regions and infected with different *Plasmodium* species were used to validate this protocol. This easy and inexpensive tool can be used for the diagnosis of *P. simium* in non-human primates and human infections from the Atlantic Forest region to monitor zoonotic malaria transmission in Brazil.

## Introduction

Zoonotic malaria constitutes a major challenge to malaria elimination. Until recently, it was thought that there were four species of malaria parasite responsible for disease in humans; *Plasmodium falciparum*, *Plasmodium vivax*, *Plasmodium malariae* and *Plasmodium ovale*. However, recent studies in Southeast Asia have demonstrated the widespread zoonotic transmission of *Plasmodium knowlesi*, a species previously thought to be confined to macaques^1^. These findings led to the recognition of *P. knowlesi* as a fifth human malaria parasite and demonstrated the zoonotic potential of non-human primate (NHP) malaria in sylvatic settings^2–4^. In addition to *P. knowlesi*, there are other *Plasmodium* species that infect NHPs and can also be transmitted to humans, such as *Plasmodium cynomolgi* in Asia and *Plasmodium brasilianum* and *Plasmodium simium* in the Americas^5–8^.

Malaria transmission in Brazil occurs almost entirely within the Amazon region^9^. However, there are a consistent number of autochthonous cases reported in Southern and Southeastern Brazil, predominantly in areas under influence of the Atlantic Forest. From 2006 to 2016, 1047 autochthonous cases were reported in these areas^9–11^. Most of these episodes are attributed to *P. vivax* and essentially affect non-resident visitors^11^. These areas are particularly rich in bromeliads, which provide a highly suitable habitat for *Kerteszia* mosquito species, especially *Anopheles Kerteszia cruzii* and *Anopheles Kerteszia bellator*^12–14^. These mosquitoes are the primary vectors of the called “bromeliad malaria”^12,15–17^. These species may bite humans and non-human primates (NHPs) with similar frequencies, depending on the area and environmental/climatic situation^16,18–20^. Based on this, zoonotic transmission has been hypothesized in these areas, with NHPs acting as a reservoir of *Plasmodium* species capable of infecting humans^5^. There are two species of malaria parasites infecting non-human primates in this region; *Plasmodium simium*, a tertian malaria and *Plasmodium brasilianum*, a quartan malaria. *Plasmodium simium* naturally infects primates of the Atelidae and Cebidae families, genera *Alouatta* (howler monkeys), *Brachyteles* (wooly spider monkeys), *Cebus* and *Sapajus* (capuchin monkeys) in South and Southeastern Brazil^21,22^. *Plasmodium brasilianum* has been detected in nearly 50 NHPs species belonging to 13 genera distributed from Panama to the South of Brazil^21,23,24^.

*Plasmodium simium* is highly similar at the morphological, genetic and immunological levels to *P. vivax*, the most prevalent human malaria parasite in Brazil^21,25,26^. Due to this, human malaria in the Atlantic Forest has previously been misdiagnosed as *P. vivax*, either by microscopy or through molecular diagnosis based on PCR of the 18S rRNA gene. However, recently, a study in the Rio de Janeiro Atlantic Forest described morphological and genetic differences (based on mitochondrial genome sequencing) between these two species, and incriminated *P. simium* as the causative agent of the great majority of human malaria cases in the region^27^. This observation was confirmed by other authors analyzing different Brazilian states under the influence of the same biome^28,29^.

As morphological discrimination is highly dependent on the subjective consideration of very well-trained microscopists, we have developed a new methodology able to identify *P. simium* samples using nested PCR followed by enzyme digestion without the need for nucleotide sequencing. This methodology relies on the fact that all known *P. simium* mitochondrial genome sequences (derived from infection of NHPs from Atlantic forest) differ from the most closely related *P. vivax* sequences (derived from infection of humans) by two unique single nucleotide polymorphisms (SNPs). This protocol can be used for the screening of the local NHPs and humans malaria cases during surveillance of zoonotic malaria transmission in the Brazilian Atlantic Forest.

## Methods

### Non-human primate samples from the Atlantic Forest

Sixteen *P. simium* and two *P. brasilianum* infected whole blood samples were obtained from captive and free-living NHPs from the Brazilian Atlantic Forest of Rio de Janeiro and Santa Catarina States (Additional file 1)^22,24^. Animals used in this study were captive primates from the Primate Centre of Rio de Janeiro (CPRJ) (n = 2) and from Biological Research Centre of Indaial (CEPESBI) (n = 7) and free living NHPs from Santa Catarina State (n = 6) and Rio de Janeiro State (n = 1). The CPRJ (Brazilian Institute of Environment and Renewable Natural Resources-IBAMA, register number 458460) is a unit for wild monkey protection. It is geographically located between latitude 22°7′ and 22°32′ S and longitude 42°50′ and 42°56′ W in the municipality of Guapimirim (one of the municipalities where human malaria occurs in the Rio de Janeiro Atlantic Forest), on the Serra dos Órgãos slopes, in an area of the Atlantic Forest, about 100 km from the city of Rio de Janeiro. Serra dos Órgãos is part of the Serra do Mar, a large coastal mountain chain in southeast Brazil. As the handling of NHPs was exclusively performed by CPRJ technicians, the Fiocruz Animal Ethics Committee agreed to the protocol for sample collection. Captive Southern brown howler monkeys (*Alouatta guariba clamitans)* from CEPESBI (Indaial, SC) (IBAMA register number 197351) were also included^30^. Free-living NHPs from the Atlantic Forest in Joinville municipality, in Santa Catarina State in southern Brazil, were obtained from a behavioral study of *Alouatta g. clamitans* in the Brown Howler monkeys conservation program (Perini Business Park). The Brazilian government authorized this study and the access to and transport of biological samples through Biodiversity Information and Authorization System (SISBIO) no. 43375-4/2015. *P. simium* DNA extracted from the spleen and liver of a single *Alouatta g. clamitans* found dead in Guapimirim was used as positive control in the PCR because it was previously sequenced by us^22^. Capture, handling and blood sampling of free living primate in Rio de Janeiro State were approved by the SISBIO licenses 54707-137362-2 and 52472-1, and INEA license 012/2016012/2016) and the Institutional Ethics Committee of animal use (CEUA license L037/2016). All experiments were performed according to relevant guidelines and regulations.

### Human DNA samples from the Atlantic Forest

Whole blood samples from patients with symptoms suggestive of malaria were used for DNA extraction. Patients were selected based on history of travel to or habitation in areas within the Atlantic Forest and a positive test by thick blood smear or PCR. The following exclusion criteria were applied: malaria prophylaxis, previous history of malaria, blood transfusion or tissue/organ transplantation, use of intravenous drugs, needle stick injury, residence or recreation near ports or airports or travel to known malaria endemic areas in the preceding year. Cases considered here are from patients who attended the Acute Febrile Illnesses Unit of the *Instituto Nacional de Infectologia Evandro Chagas*, which integrates the Center for Malaria Research, Diagnosis and Training of Fiocruz, a reference center for malaria in the Extra-Amazonian Region for the Ministry of Health. Patients’ blood samples from Atlantic Forest area diagnosed as positive for *P. vivax* by optical microscopy or PCR^31^ were included here (Additional file 2).

### DNA extraction

DNA was extracted from whole blood using the QIAGEN Kit (PUREGENE®, Gentra Systems, Minneapolis, USA) according to the manufacturer’s protocol. The DNA was diluted in 30 μl of TE Buffer and stored at −20 °C until used in the experiments.

### Human DNA samples from Amazon region

Samples of three *Plasmodium* species from patients previously diagnosed by microscopy and nested PCR^32^ were utilized to test the specificity of the novel *P. simium* primers: 2 *P. falciparum*, 15 *P. vivax* and 2 *P. malariae*, besides 2 malaria negative samples (Additional file 2). The *P. vivax* DNA samples selected for use in this study were from different parts of Amazon (Amazonas, Rondônia, Pará and Acre states of Brazil, Venezuela, French Guiana and Guyana) and were stored at the biorepository of Laboratory of Malaria at IRR.

### Ethical clearance for human samples

The Ethics and Research Committee of INI-IPEC and Fiocruz Ethical Committee in Research approved the study (No. 0062.0.009.000-11). The methods used for human DNA collection were performed according to relevant guidelines and regulations. All participants and/or their legal guardians provided written informed consent.

### Nested PCR and RFLP

Primer pairs for the nested PCR are: first reaction - *PsimOUTF* 5′CAGGTGGTGTTTTAATGTTATTATCAG3′ (forward) and *PsimINR* 5′ATGTAAACAATCCAATAATTGCACC3′ (reverse); and second reaction - *PsimEDF* 5′ATCCTACATTTGCTGGAGATCCTA3′ (forward) and *PsimEDR* 5′GCTCTTGTATCTACTTCTAAACCTGTAG3′ (reverse). The first reaction was performed in 20 μL volumes containing 0.5 μM of each oligonucleotide primer, 2 μL DNA, 0.2 μL Taq DNA Polymerase (Invitrogen, 5U/μL), 0.2 mM each deoxyribonucleotide triphosphates and 1.5 mM MgCl_2_. The PCR assays were performed in a thermocycler (Veriti 96 wells, Applied Biosystems) with the following cycling parameters: an initial denaturation at 94 °C for 2 min followed by 40 cycles of denaturation at 94 °C for 30 sec, annealing at 54 °C for 20 sec and extension at 72 °C for 30 sec followed by a final extension incubation at 72 °C for 2 min. The temperature was then reduced to 4 °C until the samples were taken. For the second PCR 1–3 μL of the primary product was used as template. The cycling parameters for the second round of PCR were the same as the first round. The amplified fragments were visualized by electrophoresis on 2% agarose gels in 1x TAE buffer (40 mM Tris-acetate, 1 mM EDTA) with 5 μg/mL ethidium bromide (Invitrogen) in a horizontal system (Bio-Rad) at 100 V for 30 min. Gels were examined with a UV transilluminator (UVP - Bio-Doc System it).

To prevent cross-contamination, the DNA extraction and mix preparation were performed in “parasite DNA-free rooms” distinct from each other. Furthermore, each of these separate areas has different sets of pipettes and all procedures were performed using plugged pipette tips. DNA extraction was performed twice on different days. Positive (DNA extracted from blood from patients with known *P. vivax* infection) and negative (no DNA and DNA extracted from individuals who have never traveled to malaria-endemic areas) controls were used in each round of amplification. The sources of genomic DNA samples that served as positive controls in the nested PCR assays are: i) *P*. *falciparum* DNA, strain 3D7 maintained in Malaria Laboratory (IRR-Fiocruz MINAS); (ii) DNA of extracted from the blood of a patient with high parasitemia for *P*. *vivax* and DNA of *P*. *simium* of a non-human primate with an acute infection and parasitemia confirmed by optical microscopy; (iii) DNA of *P. brasilianum* from the MR4 (ATCC, USA).

Following amplification (verified by agarose gel electrophoresis), PCR products were digested with the restriction enzyme *Hpy*CH4III (New England biolabs, Ipswich, MA, USA). The restriction enzyme profile was identified using NEBcutter^33^. The digestion was performed in 10 μL containing 0.5 μL of the enzyme (5U/μL), 1 μL of the enzyme’s buffer and 5 μL of PCR product (2–7 μL according to the intensity of PCR products on agarose gels). The digestion was incubated at 37 °C for 3 hours. All digestion reaction and the equivalent amount of non-digested DNA were visualized in electrophoresis on 3% agarose gel and examined under a UV transilluminator. Electrophoresis was performed in a room specific for amplified DNA, with appropriated sets of pipettes and plugged pipettes tips. For some samples, it was necessary to modify the amount of DNA in the PCR, PCR product in the second reaction or PCR product used in the digestion.

### Limits of detection of PCR amplification

The analytical sensitivity of the assay was determined using a well-quantified parasitemia of *P. simium* obtained from a patient infected in the Atlantic Forest. The percent parasitemia was determined by Giemsa’s solution-stained blood smears. To estimate parasite density per μL of blood, a standard mean WBC count of 6,000/μL blood was assumed. All slides were subsequently examined by an independent malaria microscopist qualified by the PAHO/WHO malaria accreditation course.

The resulting parasitemia was determined to be nearly 100 parasites/μL in this patient. DNA was extracted using 300 μL of blood sample. This standard DNA sample was then serial diluted two-fold to 1.65 parasites/μL. These diluted DNA samples were used to test the limits of detection of the novel *P. simium* primers set described here.

### DNA Sequencing

1–2 μL of PCR products were amplified using 5 μM of forward or reverse species-specific primer (*PsimEDF* or *PsimEDR*), 0.5 μL of BigDye terminator and 1 μL of BigDye Buffer (Applied Biosystems) for DNA sequencing. In addition, PCR products were sequenced using the primers *PsimINF* 5′ GCTGGAGATCCTATTTTATATCAAC 3′ (forward) or *PsimINR* 5′ATGTAAACAATCCAATAATTGCACC 3′ (reverse), with the same PCR conditions, resulting in sequences that covered both SNPs we consider diagnostic of *P. simium*. The following cycling parameters were used in both situations: 96 °C for 1 min, 35 cycles of 96 °C for 15 sec, followed by the temperature of primer annealing (54 °C) for 15 sec and 60 °C for 4 min. The fragments were precipitated using ammonium acetate, suspended in formamide HI-DI (Applied Biosystems) and electrophoretically separated in ABI 3730 DNA automatic sequencer. Sequences were aligned using ClustalW software in Bioedit package^34^ and Chromas software^35^.

## Results

### *Plasmodium simium* primer design

*Plasmodium simium* whole mitochondrial genome sequence (accession numbers AY800110, NC_007233 and AY722798, all of which have identical sequences) was compared with 794 *P. vivax* mitochondrial genome sequences deposited in Genbank^27^. *Plasmodium simium* differs from the most closely related *P. vivax* at two unique single nucleotide polymorphisms (SNPs) in the mitochondrial genome, at positions 3535 (T>C) and 3869 (A>G) according to the nucleotide numbering system employed in Brasil *et al*.^27^. Restriction enzyme profiling of the fragment containing these two polymorphisms was performed using NEBcutter. Restriction enzyme *Hpy*CH4III recognizes the site ACNGT which includes one of the two *P. simium* specific SNPs. The “**T**” in *P. vivax*, as well in all other *Plasmodium* species tested, is substituted by a “**C**” in *P*. *simium* (position 3535). This polymorphism adds a new restriction site for *Hpy*CH4III. Based in the alignment of this region, primers were manually designed for a nested PCR to increase the sensitivity according to the expected low parasitemia. Primer candidates were screened for GC-content, melting temperature, secondary structure, and primer-dimer forming potential. The best pairs were selected with an amplified region of 244 bp, the *P. simium* type of which being digested by *Hpy*CH4III to two bands, one 118 bp and the other 126 bp.

### Standardization of Nested/PCR-RFLP

In order to optimize the Nested/PCR, different annealing temperatures from 45 °C to 60 °C were tested. Primer concentrations from 0.5 mM to 1.0 mM were evaluated at 1.5 mM MgCl_2_ concentration (data not shown). Optimal amplification was obtained with the use of 54 °C annealing temperature, with 0.5 mM of primer. Amplified products (verified in agarose gel) were digested using the restriction enzyme *Hpy*CH4III.

### Differential diagnosis of *P. simium* using Nested/PCR – RFLP

**Figure 1:**
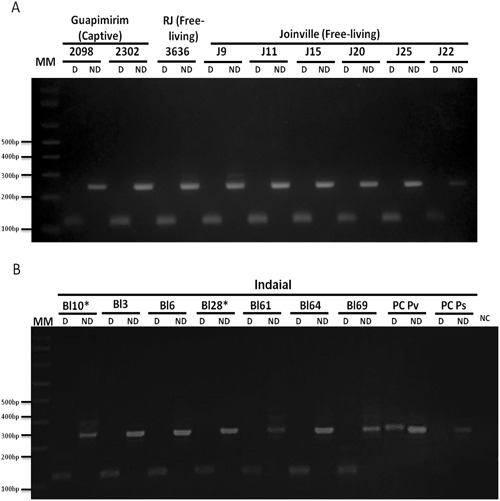
Differential Diagnosis of *Plasmodium simium* infection by nested/PCR followed by a digestion with *Hpy*CH4III restriction enzyme of 16 non-human primate samples: (**A**) 2 captive NHP from Rio de Janeiro/RJ (*Sapajus xanthosternos* 2098; *Cacajao melanocephalus* 2302), one free-living *Alouatta g. clamitans* from Rio de Janeiro State (3636) and 6 free-living *Alouatta g. clamitans* from Joinville/SC, Brazil (J9, J11, J15, J20, J22, J25); (**B**) 7 *Alouatta g. clamitans* from CEPESBI, Indaial, SC, Brazil (Bl3, Bl6, Bl10, Bl28, Bl61, Bl64, Bl69), *Captive NHPs, all the other were free-living. More details about each sample see Additional file 1. 3% agarose gel stained with ethidium bromide. MM:1 kb Plus Ladder (ThermoFischer). Reactions were performed simultaneously in the same thermocycler and splited in different gels. PC Pv: Positive Control for *P. vivax*, PC P!s: positive control for *P. simium*. NC: Negative Control (without DNA).

**Figure 2:**
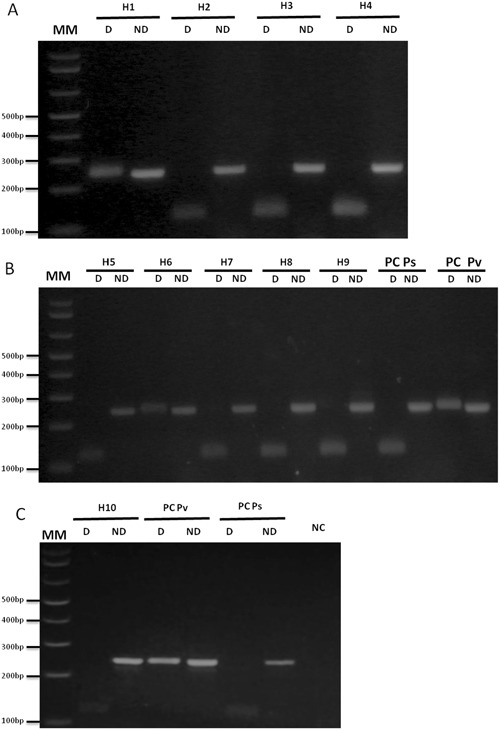
Differential diagnosis of *Plasmodium simium* infection by nested/PCR followed by a digestion with *Hpy*CH4III restriction enzyme of 9 infected human samples from Atlantic Forest in Rio de Janeiro/RJ (H2 – H9) and one from Amazon endemic region (H1) according to Additional file 1. 3% agarose gel stained with ethidium bromide. MM:1 kb Plus Ladder (ThermoFischer, Calrsbad, CA, USA). Reactions were performed simultaneously in the same thermocycler and splited in different gels.D: Digested; ND: Non Digested. PC Pv: Positive Control for *P. vivax*, PC Ps: positive control for *P. simium*. NC: Negative Control (without DNA).

**Figure 3:**
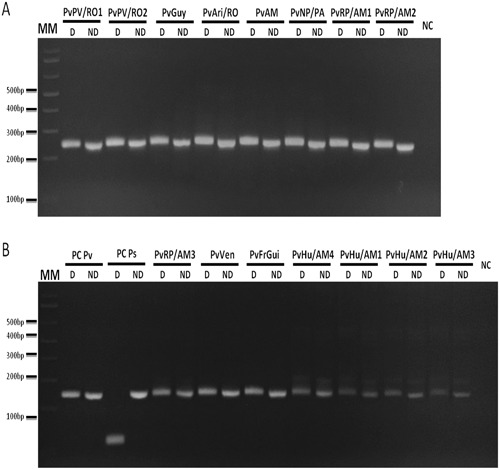
Nested/PCR-RFLP of *P. vivax* DNA samples. 15 DNA samples from *P. vivax* infected individuals from different parts of Amazon: Porto Velho/Rondônia State, Brazil (PvPV/RO1 and 2), Guyana (PvGuy), Ariquemes/Rondônia State, Brazil (PvAri/RO), Venezuela (PvVen), French Guiana (PvFrGui), Novo progresso/Pará State, Brazil (PvNP/PA), Rio Pardo/Amazonas State, Brazil (PvRP/AM1, 2 and 3), Humaita/Amazonas State, Brazil (PvHu/Am1, 2, 3 and 4) and unknown city in Amazonas State, Brazil (PvAM) were used for Nested PCR amplification followed by digestion with *Hpy*CH4III restriction enzyme. 3% Agarose gel stained with ethidium bromide. Name tags above gels indicated the patients according to Additional file 2. Reactions were performed simultaneously in the same thermocycler and splited in different gels. MM: 1 kb Plus Ladder. D: Digested (8 μL of digestion); ND: Non Digested (the equivalent amount of PCR product used in the digestion, 6.5 μL of samples and 5 μL of controls); PC Pv: positive control of *P. vivax* (pool of samples from infected patient from Amazonia); PC Ps: positive control of *P. simium* (*Alouatta g. clamitans* infected with *P. simium* previously sequenced^30^); NC: negative Control.

**Figure 4:**
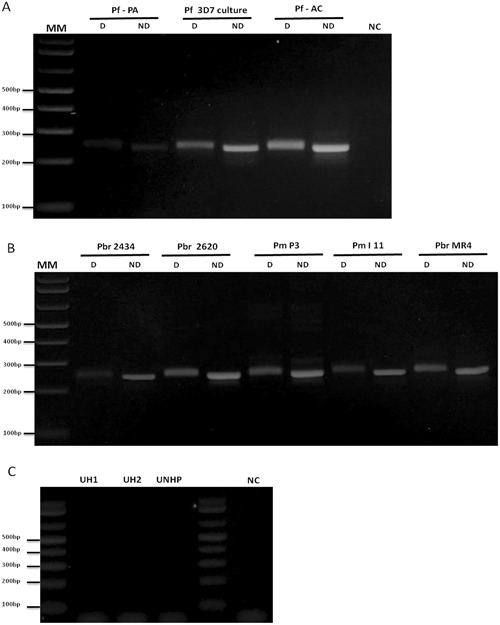
Nested/PCR-RFLP of (**A**) *Plasmodium falciparum* samples, two patients from Pará State (Pf – PA) and Acre State (Pf – AC) and Pf 3D7 culture (diluted 1:100); (**B**) *Plasmodium brasilianum/Plasmodium malariae*, two NHPs (Pbr 2434 and Pbr 2620), two patients (Pm P3 and Pm l11) and *Plasmodium brasilianum* from MR4 (diluted 1:100 in water); (**C**) Negative controls of PCR: two uninfected humans (UH - 1 and 2), one uninfected NHP (UNHP), and negative control of PCR (NC - without DNA). Reactions were performed simultaneously in the same thermocycler and splited in different agarose gels. 3% Agarose gelstained with ethidium bromide. MM: 1 kb Plus Ladder. D: Digested; ND: Non Digested.

Samples from NHPs infected by *P. simium* were analyzed using the standardized protocol. Captive and free living NHPs were from 3 different areas and samples were collected at different times (additional file 1). All 16 samples showed the digestion profile characteristic of *P. simium* (Fig. 1). Also, 8 from 9 humans infected in the Atlantic Forest showed this profile (Fig. 2). *Plasmodium vivax* from different areas of Amazon showed the profile of absence of digestion (Fig. 3). To confirm that the absence of digestion was not due to low levels of DNA, increased amounts of PCR product from three *P.vivax*-infected patients were used in the digestion with no digestion observed (supplementary Fig. S1). Similarly, DNA from other *Plasmodium* species (*P. falciparum*, *P. brasilianum/P.malariae*) were amplified by PCR but were not digested by *Hpy*CH4III (Fig. 4A and B). DNA extracted from the blood of uninfected humans, uninfected non-human primate and non-DNA solutions were included as negative controls in Nested/PCR (Fig. 4C).

### Sequencing of PCR products

**Figure 5:**
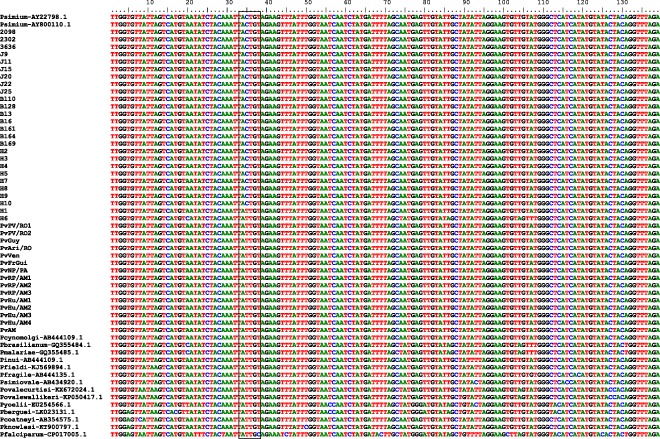
Alignment of partial mitochondrial sequences of *Plasmodium simium* isolated from captive (2098, 2302, 3636, BL10 and BL28) and free living NHPs (BL3, BL6, BL61, BL64, BL69, J9, J11, J15, J20, J22 and J25) from Atlantic forest; humans (H2, H3, H4, H5, H7, H8, H9 and H10) infected with *P. simium* at Atlantic Forest; human samples obtained in Atlantic Forest infected with *P. vivax* (H1 and H6); *P. vivax* isolated from human from Brazilian Amazonia: (PvPV/RO1 and PvPV/RO2 (Porto Velho, Rondonia), PvGuy (Guyana), PvAri/RO (Ariquimedes, Rondônia), PvVen (Venezuela), PvFrGui (French Guiana), PvNP/PA (Novo Progresso, Pará), PvRP/AM1- PvRPAM3 (Rio Pardo, Amazonia), PvHu/AM1 - PvHu/AM4 (Humaita, Amazonia) and PvAM (Amazonia State). These sequences were identified herein. Genbank sequences from *P. simium* (two sequences), *P. cynomolgi*, *P. inui*, *P.fieldi*, *P. fragile*, *P. coatneyi*, *P. simiovale*, *P. berguei*, *P. falciparum*, *P. ovale curtisi*, *P. ovale wallikeri*, *P. yoelii* and *P. knowlesi* (accession number at genbank included in the name of sequence). Box delimited the site of *Hpy*CH4III restriction enzyme (ACNGT), including SNP T>C at position 3535^27,28^.

Products of Nested/PCR were sequenced. Sequences obtained were aligned with sequences available at GenBank (Fig. 5). The *P. simium* specific SNP was confirmed in the majority of Atlantic Forest samples, including all NHPs and 8 out 9 of human-derived samples. In addition, all samples were subjected to PCR and sequencing of a larger product that included both potentially diagnostic SNPs. All samples that contained the 3535 (T>C) SNP also carried the 3869 (A>G) SNP, and no hybrids carrying one or the other SNP in isolation were observed (Fig. S2).

### Limits of detection of the Nested PCR

**Figure 6:**
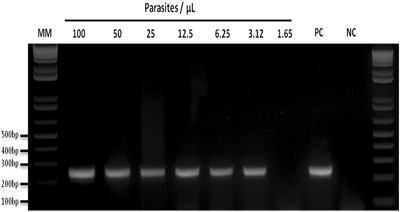
Detection limit of the Nested-PCR for a differential diagnosis of *Plasmodium simium. P. simium* human DNA sample were serially diluted 2-fold with a starting parasitemia of 100 parasites/µL to 1.65 parasites/µL. 2% Agarose gel stained with ethidium bromide. MM:1 kb Plus Ladder, PC: positive control, NC: negative control (without DNA).

Limits of detection of the Nested-PCR as were tested with *P. simium* DNA extracted from an infected human serially diluted two-fold with a starting parasitemia of 100 parasites/μL to 1.65 parasites/μL. The novel Nested PCR assay was able to detect down to 3.12 parasites/μL (Fig. 6).

## Discussion

Zoonotic transmission of *Plasmodium* spp. complicates the control of malaria as NHPs can act as reservoirs. The process of urban expansion potentially increases contact between free-living NHPs and humans, as towns and cities encroach on previously untouched forested areas. There is a large human population worldwide currently living in the close vicinity of forests where many different species of NHPs potentially carry *Plasmodium* spp.

Neotropical Platyrrhine primates are distributed from Mexico to Argentina. To date, *P. simium* has been described only in the Atlantic Forest of South and Southeastern Brazil. However, it is possible that this species might also be prevalent in other biomes in South America, and that its zoonotic transmission could be a much larger problem than currently recognized. Herein we described an inexpensive tool for the discrimination of *P. vivax* from the agent we currently consider to be *P. simium*.

Only two unique single nucleotide polymorphisms (3535 T>C and 3869 A>G) in the whole mitochondrial genome sequence differentiate *P. simium* from *P. vivax*^27,28^. All 16-monkey samples from 3 different areas of Brazilian Atlantic Forest show both these polymorphisms. Up to now, there has not been a single case of a parasite detected in a NHP in Brazil which did not harbor the *P*. *simium* version of these SNPs. Every single *P. vivax*-like parasite found in New World NHPs carries it, including the ‘type’ *P*. *simium* strain deposited at the US Centers for Disease Control and Prevention (CDC) repositories^36^.

Buery and colleagues have recently reported the presence of the two *P. simium* SNPs in one monkey sample and in 17 of 22 human infections from the Atlantic Forest area^29^.Two out of the 22 infections carried *P. vivax* versions of the SNPs at both loci. It is possible that these latter cases are due to imported *P. vivax* from Amazonia. However, they also found 3 of the 22 Atlantic Forest-human samples harbouring only one of the *P. simium-*specific SNPs that have not previously been observed in isolation. It is possible that these latter samples are the result of mixed infections (*P. vivax* and *P. simium*) or are *P. vivax* variants that do not infect NHPs (as these haplotypes have never been observed in NHPs). It is unlikely that these infections were caused by *P. simium* variants because of the low genetic variability of this parasite^27,28,30^. Espirito Santo State has a history of intense migration to Amazon area, where *P. vivax* is endemic^37^, and it may be expected that both parasites circulate in this area due to the presence of different anopheline species^38^. However any firm conclusions concerning the origins and relatedness of parasites in this study are hampered by the small number of samples collected from NHPs (n = 1), and differing times and locations of collection of samples.

It is possible that “*P. simium*” infecting NHPs in Brazil is, in fact, a variant strain of the human parasite *P. vivax*, and does not constitute a separate species based on a strict biological definition. It is possible that what we now consider to be *P. simium* and *P. vivax* are actually one and the same species, variants of which circulate freely between humans and NHPs in the Atlantic Forest when vectors and ecology allow. It will only be possible to conclusively address this issue with thorough surveillance of parasites from NHPs, and whole genome sequencing data. However, whether we consider *P. simium* a separate species, a sub-species or a strain *of P. vivax* does not alter the epidemiology and public health consequences of a monkey harbored parasite that is able to infect humans.

The *P. simium* specific SNP that forms the basis of our diagnostic method is carried by all parasites isolated from NHPs so far. It was also observed in the majority (8 out 9) of Atlantic Forest human samples. The other human case was a *P. vivax* infection that might be due to an undetected index case from Amazon area. No other *Plasmodium* strain or species has this polymorphism, as show in Fig. 5. We can be reasonably certain, in the absence of evidence to the contrary, that this SNP is exclusive to *P. simium*, and can be used to distinguish this zoonotic species from other species of malaria parasite. The novel Nested PCR assay was able to detect ≥3.12 parasites/μL, which is similar to the detection limit of the most used 18S-based PCR protocol in our laboratory^30,32^.

In conclusion, we show that humans and NHPs in the Atlantic Forest region harbor the same parasite. We, therefore, confirm our previous findings showing that NHPs constitute a potential zoonotic reservoir, now with a greater number of samples, and conclude that zoonotic transmission is probably occurring in this biome^27^. The assay described here will, therefore, aid the surveillance of zoonotic malaria transmission in Brazil. Further sampling from NHPs, and further genetic characterization of their parasites and those from humans in the same region will further elucidate the relationships between the parasites circulating between different host species in the Atlantic Forest as well in regions of Brazil and South America.

### Data availability

All data provided in this manuscript is available for open access.

## Electronic supplementary material

Supplementary information
