# Author Correction: An assay for the identification of *Plasmodium simium* infection for diagnosis of zoonotic malaria in the Brazilian Atlantic Forest

**DOI:** 10.1038/s41598-019-53954-0

**Published:** 2019-11-21

**Authors:** Denise Anete Madureira de Alvarenga, Richard Culleton, Anielle de Pina-Costa, Danielle Fonseca Rodrigues, Cesare Bianco, Sidnei Silva, Ana Júlia Dutra Nunes, Julio César de Souza, Zelinda Maria Braga Hirano, Sílvia Bahadian Moreira, Alcides Pissinatti, Filipe Vieira Santos de Abreu, André Luiz Lisboa Areas, Ricardo Lourenço-de-Oliveira, Mariano Gustavo Zalis, Maria de Fátima Ferreira-da-Cruz, Patricia Brasil, Cláudio Tadeu Daniel-Ribeiro, Cristiana Ferreira Alves de Brito

**Affiliations:** 1 Grupo de Pesquisa em Biologia Molecular e Imunologia da Malária, Instituto René Rachou (IRR), Fundação Oswaldo Cruz (Fiocruz), Belo Horizonte/MG, 30190-009 Brazil; 2 Malaria Unit, Department of Pathology, Institute of Tropical Medicine, Nagasaki University, Nagasaki, 852-8523 Japan; 3 Laboratório de Doenças Febris Agudas, Instituto Nacional de Infectologia Evandro Chagas (INI), Fiocruz, Rio de Janeiro/RJ, 21040-360 Brazil; 4 Centro de Pesquisa, Diagnóstico e Treinamento em Malária (CPD-Mal), Fiocruz, Rio de Janeiro/RJ, 21040-360 Brazil; 5 Laboratório de Pesquisa em Malária, Instituto Oswaldo Cruz (IOC), Fiocruz, Rio de Janeiro/RJ, 21040-360 Brazil; 6 Núcleo de Educação Ambiental Fábio Perini, Perini Business Park, Joinville/SC, 89219-600 Brazil; 7 Centro de Pesquisas Biológicas de Indaial, Indaial/SC, 89130-000 Brazil; 8 Universidade Regional de Blumenau – FURB, Blumenau/SC, 89012-900 Brazil; 9 Centro de Primatologia do Rio de Janeiro (CPRJ/INEA), Guapimirim/RJ, 25940-000 Brazil; 10 Centro Universitário Serra dos Órgãos (UNIFESO), Teresópolis/RJ, 25964-004 Brazil; 11 Laboratório de Mosquitos Transmissores de Hematozoários, Fiocruz, Rio de Janeiro/RJ, 21040-360 Brazil; 12 Laboratório de Infectologia e Parasitologia Molecular, Hospital Universitário Clementino Fraga Filho, Universidade, Federal do Rio de Janeiro/RJ, 21941-901 Brazil

Correction to: *Scientific Reports* 10.1038/s41598-017-18216-x, published online 08 January 2018

The original version of this Article omitted an affiliation for Anielle de Pina-Costa. The correct affiliations for Anielle de Pina-Costa are listed below:

Laboratório de Doenças Febris Agudas, Instituto Nacional de Infectologia Evandro Chagas (INI), Fiocruz, Rio de Janeiro/RJ, 21040-360, Brazil

Centro de Pesquisa, Diagnóstico e Treinamento em Malária (CPD-Mal), Fiocruz, Rio de Janeiro/RJ, 21040-360, Brazil

Laboratório de Pesquisa em Malária, Instituto Oswaldo Cruz (IOC), Fiocruz, Rio de Janeiro/RJ, 21040-360, Brazil

Centro Universitário Serra dos Órgãos (UNIFESO), Teresópolis/RJ, 25964-004, Brazil

This has now been corrected in the HTML and PDF versions of this Article, and in the accompanying Supplementary Information.

