# Supplementary material for: An assay for the identification of Plasmodium simium infection for diagnosis of zoonotic malaria in the Brazilian Atlantic Forest
Source: Sci Rep. 2018 Jan 8;8:86. doi: 10.1038/s41598-017-18216-x (PMC5758784; doi:10.1038/s41598-017-18216-x)
Supplement: Supplementary file 1 — Supplementary information [file 41598_2017_18216_MOESM1_ESM.docx]

**Supplementary information**

**An assay for the identification of *Plasmodium simium* infection for diagnosis of zoonotic malaria in the Brazilian Atlantic Forest**

*Denise Anete Madureira de Alvarenga ^1#^, Richard Culleton^2#^, Anielle de Pina-Costa^3,4,5,10^, Danielle Fonseca Rodrigues^1^, Cesare Bianco Júnior^,4,5^, Sidnei Silva^3^, Ana Júlia Nunes*^6^*, Julio César de Souza Jr^7,8^, Zelinda Hirano^7,8^, Sílvia Bahadian Moreira^9^, Alcides Pissinatti^9,10^, Filipe Vieira Santos de Abreu^11^, André Luiz Areas^12^, Ricardo Lourenço de Oliveira^11^, Mariano Zalis^12^, Maria de Fátima Ferreira-da-Cruz^4,5^, Patricia Brasil^3^, Cláudio Tadeu Daniel-Ribeiro^4,5^, Cristiana Ferreira Alves de Brito^1*^*

Table S1. Description of non-human primates samples

Table S2. Description of human samples

Fig S1. Restriction enzyme digestion performed using different amounts of DNA. DNA of three *P. vivax* infected patients from: Porto Velho/Rondonia (PvPV/RO1)(lanes 1-4), Novo Progresso/ Pará (PvNP/PA) (lanes 5-8) and Rio Pardo/Amazonia (PvRP/AM1) (lanes 9-12) were used in Nested PCR. Different amounts of PCR products were used in the restriction enzyme digestion: 5 µL (lanes 1, 5 and 9), 6.5 µL (lanes 2, 6 and 10) and 8.5 µL (lanes 3, 7 and 11), compare to 5 µL of undigested PCR products (lanes 4, 8 and 12). As positive controls were used DNA from *P. simium* infected NHP previously sequenced^22^ (PC Ps) and pool of *P. vivax* infected patients from Amazonia area (PC Pv), 5 µL were used in the digestion (lanes 13 and 15) or undigested directly applied on gel (lanes 14 and 16). 3% Agarose gel stained with ethidium bromide. MM: 1 kb Plus Ladder. PC: Positive Control and NC: Negative Control (absence of DNA on ).

**Fig S2.** Alignment of partial mitochondrial sequences including both SNPs (T>C at position 3535^27,28^, here position 48; and A>G at position 3869^27,28^, here position 382)of *Plasmodium* *simium* isolated from captive (2098, 2302, 3636, BL10 and BL28) and free living NHPs (BL3, BL6, BL61, BL64, BL69, J9, J11, J15, J20, J22 and J25) from Atlantic forest; humans (H2, H3, H4, H5, H7, H8, H9 and H10) infected with *P. simium* at Atlantic forest; human from Atlantic forest infected with *P. vivax* (H1 and H6); *P. vivax* isolated from human from Brazilian Amazonia: (PvPV/RO1 and PvPV/RO2 (Porto Velho/Rondonia), PvGuy (Guyana), PvAri/RO (Ariquimedes, Rondonia), PvVen (Venezuela), PvFrGui (French Guiana), PvNP/PA (Novo Progresso, Pará), PvRP/AM1- PvRPAM3 (Rio Pardo, Amazonia), PvHu/AM1 - PvHu/AM4 (Humaita, Amazonia) and PvAM (Amazonia State); human infected with *P. falciparum* (1731); human infected with *P. malariae* (I11); NHP infected with *P. brasilianum* (Pbr 2620). These sequences were identified herein. Genbank sequences from *P. simium* (two sequences), *P. cynomolgi* (12 sequences), *P. inui*, *P.fieldi*, *P. fragile*, *P. coatneyi*, *P. simiovale*, *P. berguei*, *P. falciparum*, *P. ovale curtisi*, *P. ovale wallikeri*, *P. yoelii* and *P. knowlesi*. Box delimited the restriction enzyme site (ACNGT).
